# A call for action to establish a research agenda for building a future health workforce in Europe

**DOI:** 10.1186/s12961-018-0333-x

**Published:** 2018-06-20

**Authors:** Ellen Kuhlmann, Ronald Batenburg, Matthias Wismar, Gilles Dussault, Claudia B. Maier, Irene A. Glinos, Natasha Azzopardi-Muscat, Christine Bond, Viola Burau, Tiago Correia, Peter P. Groenewegen, Johan Hansen, David J. Hunter, Usman Khan, Hans H. Kluge, Marieke Kroezen, Claudia Leone, Milena Santric-Milicevic, Walter Sermeus, Marius Ungureanu

**Affiliations:** 1 Institut für Epidemiologie, Sozialmedizin und Gesundheitssystemforschung, Medizinische Hochschule Hannover, OE 5410, Carl-Neuberg-Str. 1, 30625 Hannover, Germany; 2 Karolinska Institutet, Medical Management Centre, LIME, Tomtebodavagen 18a, 171 77 Stockholm, Sweden; 3 Netherlands Institute for Health Services Research, Otterstraat 118-124, 3513 Utrecht, The Netherlands; 4 European Observatory on Health Systems and Policies, Place Victor Horta/Victor Hortaplein, 40/10, 1060 Brussels, Brussels Belgium; 5 Global Health and Tropical Medicine & WHO Collaborating Center on Health Workforce Policy and Planning, Institute of Hygiene and Tropical Medicine-NOVA University of Lisbon, Rua da Junqueira 100, 1349-008 Lisbon, Portugal; 6 Department of Healthcare Management, Technische Universität Berlin, Strasse des 17. Juni 135, 10623 Berlin, Germany; 7 Department of Health Services Management, Faculty of Health Science & WHO Collaborating Centre for Health Systems and Policy in Small States at the Islands and Small States Institute, University of Malta, Msida, MSD 2080 Malta; 8 European Public Health Association (EUPHA), Utrecht, Netherlands; 9 Christine Bond, Institute of Applied Health Sciences, University of Aberdeen, Foresterhill, Aberdeen, AB25 ZD United Kingdom; 10 Department of Public Health, University of Aarhus, Bartholins Allé 2, 8000 Aarhus C, Denmark; 11 ISCTE-Instituto Universitário de Lisboa, School of Sociology and Public Policies, Avenida das Forcas Armadas, 1649-026 Lisbon, Portugal; 12 Department of Sociology, Utrecht University, Heidelberglaan 2, 3584 CS Utrecht, The Netherlands; 13 Department of Human Geography, Utrecht University, Heidelberglaan 2, 3584 CS Utrecht, The Netherlands; 14 Institute of Health and Society, Newcastle University, Newcastle, United Kingdom; 15 European Health Management Association (EHMA), Rue Belliard 15-17, 1040 Brussels, Belgium; 16 Division of Health Systems and Public Health, WHO Regional Office for Europe, Marmorvej 51, 2100 Copenhagen, Denmark; 17 Department of General Practice, Erasmus University Medical Center Rotterdam, Wytemaweg 80, 3015 CN Rotterdam, The Netherlands; 18 Florence Nightingale Faculty of Nursing and Midwifery, King’s College London, 57 Waterloo Road, SE1 8WA London, United Kingdom; 19 Institute of Social Medicine, Faculty of Medicine University of Belgrade, Dr Subotica, Belgrade, 15 11000 Serbia; 20 KU Leuven Institute for Healthcare Policy, Kapucijnenvoer 35 blok d – box 7001, 3000 Leuven, Belgium; 21 Department of Public Health, College of Political, Administrative and Communication Sciences, Babeș-Bolyai University, 7 Pandurilor Street, 400376 Cluj-Napoca, Romania

**Keywords:** Health workforce research, Health workforce policy, Health workforce governance, Human resources for health, Health professions, Skill mixes, Integrated care, Health workforce mobility, Europe

## Abstract

The importance of a sustainable health workforce is increasingly recognised. However, the building of a future health workforce that is responsive to diverse population needs and demographic and economic change remains insufficiently understood. There is a compelling argument to be made for a comprehensive research agenda to address the questions. With a focus on Europe and taking a health systems approach, we introduce an agenda linked to the ‘Health Workforce Research’ section of the European Public Health Association. Six major objectives for health workforce policy were identified: (1) to develop frameworks that align health systems/governance and health workforce policy/planning, (2) to explore the effects of changing skill mixes and competencies across sectors and occupational groups, (3) to map how education and health workforce governance can be better integrated, (4) to analyse the impact of health workforce mobility on health systems, (5) to optimise the use of international/EU, national and regional health workforce data and monitoring and (6) to build capacity for policy implementation. This article highlights critical knowledge gaps that currently hamper the opportunities of effectively responding to these challenges and advising policy-makers in different health systems. Closing these knowledge gaps is therefore an important step towards future health workforce governance and policy implementation. There is an urgent need for building health workforce research as an independent, interdisciplinary and multi-professional field. This requires dedicated research funding, new academic education programmes, comparative methodology and knowledge transfer and leadership that can help countries to build a people-centred health workforce.

## Background

The health workforce is central to health system performance and to population health, and is critical to quality of care and patient safety. Additionally, it has an enormous economic significance and directly impacts on economic growth [1], accounting for more than 10% of total employment in several Organisation of Economic Co-operation and Development (OECD) countries [2]. Better healthcare increases the potential productive life years, reduces unemployment and productivity loss due to chronic illness, and avoids premature labour market exit, hence decreasing social benefit expenditures [2]. The importance of better healthcare policy and governance to achieve a competent and sustainable health workforce is increasingly recognised in Europe [3–10]. This is now also supported by improved data sources as well as by international recommendations and frameworks for action [1, 2, 11–16]. However, many challenges remain.

Stronger evidence-informed policies are needed as all European countries struggle to sufficiently prepare their workforce to effectively respond to socio-demographic changes and an increase in chronic non-communicable diseases (NCDs) and multi-morbidity. In many countries, the health workforce is also threatened by shortages [15, 17], and some countries have been affected by austerity politics which reduced the number of health workers employed, deteriorated working conditions, and in some cases led to emigration [18, 19]. These conditions call for population and system-specific approaches and greater attention to diversity and contexts. There are also sector-specific workforce needs to respond to the reconfiguration of hospitals, the strengthening of primary healthcare, and an increasing focus on mental health, social, long-term, and end of life care. At the same time, the inter-dependence of health systems with regards to health workforce planning calls for improved coordination and integration between countries and between national healthcare systems, sectors, providers and professional groups [20–22].

We, a group of health workforce researchers from different institutions and European countries and with different professional backgrounds, argue that there is a need for action to step-up efforts to improve health workforce governance and leadership and address knowledge gaps through research. Closing these knowledge gaps is an important step towards more effective health workforce governance and policy implementation. We introduce a complex research agenda that is closely linked to a new ‘Health Workforce Research’ section of the European Public Health Association. With a focus on Europe and taking a health system and governance approach, six major objectives have been identified as follows: (1) to develop frameworks that align health systems/governance and health workforce policy/planning, (2) to explore the effects of changing skill mixes and competencies across sectors and occupational groups, (3) to map how education and health workforce governance can be better integrated, (4) to analyse the impact of health workforce mobility on health systems, (5) to optimise the use of international/European Union (EU), national and regional health workforce data and monitoring, and (6) to build capacity for policy implementation.

In our overview below, we highlight critical knowledge gaps at different levels of health workforce governance and in different policy areas and professional groups. These persisting gaps seriously hamper the efforts of policy-makers seeking evidence-informed responses to implement health workforce governance and policy reforms more successfully. We therefore believe it is time to define health workforce research in Europe as a priority academic field within the wider health systems research agenda, bringing together knowledge and research in a systematic and comprehensive way, and developing effective problem-oriented knowledge transfer and leadership. This can ensure that policy-makers have access to the best evidence available on what the challenges are, which policy options can be envisaged, and how to increase the probability of successful implementation.

## Challenges to health workforce policy and relevant research gaps

Knowledge about concepts and tools for health workforce policy have significantly improved [6–8, 15, 23]. We also know what is driving change in the supply, need and demand for health workers, and what strategies could improve people-centred health workforce planning and governance [1, 2, 16, 24–26]. It is also increasingly recognised that workforce change goes far beyond mere numbers and shortages. The drivers are highly complex and include, among others, stronger primary care and integrated care policies which may lead to new connections between health and social care and involvement of informal carers, new technologies (eHealth, robots, etc.) and improved health literacy to respond to changing expectations of the public and strengthen user participation [11, 26].

However, understanding how to meet the new demands and needs for integration and participation in healthcare systems that are fundamentally built on ‘silo approaches’ and a hierarchical structure remains a challenge. These approaches are relevant on all levels of healthcare governance and shape the content of policy reforms, service organisation and professional practice; they are strongly embedded in the education and organisation of the health workforce. Moreover, occupational categories based on silos inform health workforce governance and planning as well as research, despite some novel approaches and European comparative research, such as the recent OECD feasibility study on skills assessment, which seeks to focus on tasks and functions rather than occupational categories [11]. Knowledge is poorly developed with regards to the policy levers for successfully implementing innovative and transformative actions and interventions, as well as the tools and channels through which to foster dissemination of this knowledge and provide advice for policy-makers. More specifically, the six major objectives previously mentioned and the critical knowledge gaps will be discussed in greater detail below.

### Develop frameworks that align health systems/governance and health workforce policy/planning

Bringing a health systems and governance approach to health workforce research is a priority goal to better understand the multi-level, multi-professional and multi-sectoral conditions of successful policy development and implementation. The recent high-level regional meeting on NCDs, led by the WHO Regional Office for Europe, has highlighted the relevance of a health systems approach: “*Workforce policy and planning, regulation and management are aligned with service planning and delivery, and support integrated teams rather than isolated individual health professionals, effectively addressing NCDs at all levels of service*” ([9], p. 6). A skills assessment by OECD has added further evidence “*for a systems-relevant approach*”, because “*the existing skills assessment instruments do not readily enable differentiation between the skills mismatch caused, on the one hand, by the inadequacies of the education and training system or, on the other hand, by the inadequacies of competing pressures in the health system*” ([11], p. 69). A recent statement for the consultation of the next EU Research and Innovation Programme on behalf of the European Public Health Association section ‘Health Workforce Research’ has additionally addressed the need for differentiation between the levels of systems, sectors, professions and individuals, and introduced suggestions for research [27].

While knowledge and tools have been improved significantly, still little is known on how to implement these tools in a variety of healthcare systems. What works, in which settings, why and how is not always well understood. Moreover, even if we know what could be done to create a more sustainable and people-centred health workforce [1, 15, 16, 24], we often do not know how to make it happen. Strategic leadership, change management, and effective knowledge transfer and brokering remain major challenges that often block successful implementation of policy reforms [28–32]. This is especially true for multi-professional environments and trans-sectoral care delivery. Currently dominant health workforce planning and governance models are still shaped by a uni-professional ‘silo’ approach, which does not take into account the evolving roles of the different professions and does not respond to changing population needs.

Another problem is that little comparative health workforce research is available that takes a broader perspective [33]. Health system research and comparative health policy is primarily built on economic indicators, especially financial expenditures [34–36], while human resources are usually either ignored or reduced to the numbers of doctors (and sometimes also nurses). Accordingly, we do not know how a successful integrated, people-centred health workforce planning model or an effective governance tool in one healthcare system plays out in another.

This brings the importance of a whole health systems approach into play, which enables the formulation of an overarching strategy to ensure higher effectiveness in health workforce policy and governance. It offers a practical way to strengthen health systems through a ‘systems thinking’ lens by making complex issues better understood. This helps to explain not only what works (or does not work), but for whom and under what circumstances [37, 38]. Further benefits of systems thinking include the capacity to promote dynamic networks of diverse stakeholders, to inspire continued learning, and to foster more system-wide planning, evaluation and research [38]. Therefore, research on the connections between health systems and governance models and the health workforce is an overarching priority issue and an important factor to enhance policy learning and translation between countries.

### Explore the effects of changing skill mixes and competencies across sectors and occupational groups

There is increasing evidence of the benefits of the professional development of nurses in new roles [39–47]. The benefits of stronger primary healthcare systems through the scaling-up of the role of general medical doctors in relation to specialists and of the delegation of tasks from doctors to nurses and other healthcare professionals, as well as better workforce planning are now also documented [9, 23, 26, 48–50]. Nevertheless, Maeda and Sotcha-Dietrich, in their recent skills assessment [11], highlighted the need for assessing tasks and functions rather than specific professional categories.

Furthermore, not enough attention has been given thus far to the composition of the health workforce, and the capacity and competencies of middle- to lower-qualified professional groups to innovate in service provision nor of the barriers that prevent them from doing so. It is also important to understand that health workforce needs may be different in hospital care, primary care, community or long-term care and in specific areas like rehabilitation [51] or public health [52]. Furthermore, there are significant differences and high variation in the composition of health workforces in Europe [33, 49, 53–56]. Whilst some of these particularities can be traced back to historical development, the underlying reasons and driving forces that create and perpetuate these differences remain largely unknown. We therefore do not know how to effectively utilise skill mixes in different healthcare systems and how to establish an approach which moves beyond the professional silos [11], underlining the need for a health systems approach [21, 27].

Furthermore, an increase in NCDs resulting in chronic conditions and in multi-morbidity [9, 10] has strengthened the demand for primary care and elder/long-term care services. A more integrated approach between health and social care is therefore a priority goal to serve the multifactorial needs of this population. However, this is one of the most under-researched areas. Research is also needed to overcome professional boundaries, to coordinate provider organisations and financing systems across sectors, and to promote the development of new skills and competencies of health professionals to effectively respond to patient needs [16, 23, 57, 58].

### Map how education and health workforce governance can be better integrated

The strengthening of education and training programmes is the foundation of future health workforce governance and a key to better prepare health professionals for people-centred and integrated, team-based care provision. Whilst many now pay lip service to this, interprofessional education is still not fully and meaningfully integrated into current educational and continuous professional development/learning programmes; instead, uni-professional competency frameworks often remain influential (e.g. [59]). Research has revealed a need for a health systems approach to transform the educational systems [60, 61]. A health systems approach directs our attention to cross-sectoral governance of health and education systems and calls for integration and coordination to overcome the ‘professional silos’ in healthcare [7, 11, 62].

Better integration is also needed in relation to the entire range of available health human resources. Efforts must be strengthened to improve the inclusion of new labour market groups, such as older health professionals, migrants and men in caring professions, and to increase the participation of women in leadership positions. For instance, Europe’s gender mainstreaming policy [63] is still poorly connected to health workforce education and research [64]. Notably, academic health centres in different EU countries show a persisting gender gap in leadership and management positions, which is bigger in academia than in hospitals [65]. Improved standardisation of health professional education across European countries [66] and an expansion of the recognition of qualifications of provider groups such as, for instance, physiotherapists, social workers and public health professionals, and non-academically qualified groups (e.g. physician/medical assistants, nursing assistants), is also important.

### Analyse the impact of health workforce mobility on health systems

Migration and mobility of health professionals have significantly increased over recent years and have rendered health systems performance subject to inter-dependencies beyond national borders. Given that mobility of persons is a founding principle of the EU, there is a special responsibility in taking the leadership in developing mechanisms to monitor the mobility of health professionals and its effects. A major challenge is to reduce inequality between EU Member States and counterbalance the risks of push-pull factors that benefit the resource-rich countries and threaten the healthcare systems in some Eastern and Southern European countries [18, 19, 67–74]. These factors are also relevant within countries and may create health workforce shortages in rural and remote areas [75].

While many recruitment and retention interventions have been developed to improve the geographical maldistribution of health professionals [17, 71], very little is known about their effectiveness. This is true for the micro-level, the experiences and deployment of healthcare workers in the destination countries [69, 76], and for macro-level policy interventions. Notably, the effects of structured mobility programmes in addressing health workforce retention and providing improved access to specialised health services across Europe remain largely unexplored [77]. The high context-dependency of recruitment and retention needs and interventions [17, 25, 75], again emphasises the need for a health systems and multi-level governance approach, that can identify facilitators and barriers in different areas.

Existing maldistribution and growing inequity concerns in the EU call for new strategies to govern mobile health workers more effectively across countries, while at the same time respecting free mobility [19]. It is also important that Europe shares a global responsibility and improves the monitoring of the international workforce flows especially from resource-poor countries. This also includes better knowledge of the recruitment strategies in resource-rich countries, the individual motivation of health workers, and the opportunities for improving equity and solidarity [19]. The Global Code of Practice on International Recruitment of Health Personnel [78] and the introduction of ‘circular migration’ in health workforce policies [8] may be useful tools to mitigate negative effects. Yet more has to be done at the EU level, for instance, greater attention to the health workforce in the European Semester [79] and other economic steering tools and structural funds; there is also a need for greater responsibility to prevent ‘brain drain’ of health workers in resource-poor countries in the global South and small islands developing states, which are particularly vulnerable to health workforce ‘brain drain’ and ‘care drain’.

### Optimise the use of international/EU, national and regional health workforce data and monitoring

Data sources are the basis of health workforce planning. Analyses have been significantly improved over recent years [12, 15], yet major problems remain. International, national and regional data sources are still poorly integrated, and the opportunities of more detailed regional data sources are not used effectively. On top of this, there remains a wide variation in indicator definitions, registration methodologies and data availability. The situation is worst when it comes to nurses, therapists and public health professionals as well as lower-level qualified occupations (e.g. nursing assistants, medical assistants), yet these are precisely the groups with high capacity for transforming service delivery. Even new titles of higher-qualified nurses may mean different things in different countries, as for instance ‘nurse practitioner’ or ‘advanced practice nurse’. Qualitative health workforce indicators are overall in a developmental stage and usually not measured, as for instance, competencies and team-based skills [20]. These conditions hamper the use of data in workforce planning and to inform policy-makers and create major problems for international comparative research [2, 15, 34].

Furthermore, planning and monitoring are primarily concerned with highly qualified, academically trained health professionals, especially doctors, and more recently also nurses, while comprehensive data on other occupational groups are often lacking [80]. Professional ‘silo’ approaches are still dominant, while little attention is paid to standardised measurements of teams and the occupational composition of the health workforce [11, 55]. This has been addressed to some degree by the most recent Joint Questionnaire and Health Workforce Account programmes of the OECD/EUROSTAT/WHO [81, 82]. Hence, the current need remains to obtain reliable data to inform integrated health workforce planning, to improve cross-country comparative approaches and to set up more comprehensive monitoring systems that are responsive to changing health workforce needs as well as to the needs of different countries. The challenge is to fully include the large and small, centralised and decentralised/federalist/community-based, and resource-rich and resource-poor countries, as well as their urban and remote/rural regions.

### Build capacity for policy implementation

The implementation of new health policies and planning models is the yardstick of capacity-building for a future health workforce, yet knowledge is particularly lacking in this area. Greater attention to the ways in which the health workforce is governed and new health policies and reform models are implemented, monitored and evaluated are therefore among the most urgent issues [20, 21, 83, 84]. This leads us back to the need for bringing a health systems and governance approach to the health workforce [9, 11, 21, 27]. One particularly important strategy is the strengthening of stakeholder involvement. A recent study of the European Observatory on Health Systems and Policy has introduced a systematic framework for assessing the outcomes of healthcare governance, which also improves the opportunities for cross-country comparison of health workforce governance. This assessment tool comprises five major dimensions, namely transparency, accountability, participation/stakeholder involvement, integrity and capacity of governance approaches – the ‘TAPIC’ framework [84].

When applied to the health workforce, strengthening participatory approaches and stakeholder involvement may help to connect bottom-up and top-down developed policy solutions, to facilitate capacity-building for integrated and sustainable health workforce planning, and to develop cost-effective monitoring systems. Stakeholder involvement may reduce sectoral fragmentation and other governance gaps at the macro-level; useful practical tools are, for instance, multisectoral planning groups, shared budgets, interprofessional learning and multi-professional networks [22, 49, 85]. However, the transformative capacity of stakeholder involvement does not come without conditions. It is strongly shaped by health system characteristics; for instance, conservative corporatist arrangements may promote the medical profession more than other health professions, and new public management may prioritise organisational interests over people-centred care [86].

Furthermore, professional groups have primarily been researched as ‘tribes’ that narrowly defend their professional boundaries and interests, and therefore counteract integrated service provision and people-centred care. Yet, the health professions are also serving patients and the public. Little attention has been paid so far to their capacity to develop new competencies and innovate service provision according to patient needs and to their role as policy experts [87]. To foster innovation in health workforce governance more systematically, a better understanding of professional stakeholder involvement – and more generally of effective governance [84, 85] – is needed.

## Conclusions

This brief overview has highlighted important knowledge gaps and introduced major objectives for health workforce policy in Europe. The knowledge gaps cannot be solved effectively by simply collecting ‘more of the same’ data. There is a need for establishing a comprehensive research agenda, which includes new comparative methodological and theoretical approaches [27], and a better understanding of leadership, implementation processes and policy levers for creating an integrated people-centred health workforce in diverse healthcare systems and sectors [1, 16]. This also highlights the importance of different institutional and political contexts, and the lack of knowledge on what works well in which context. One important step forward can be to bring a health systems approach to the health workforce, which pays greater attention to convergence and divergence of health workforce needs and explores opportunities for governance innovation and stakeholder involvement in context, and for new assessment approaches [11, 26, 84, 85].

We have also shown that critical knowledge gaps exist at different levels of health workforce governance and in different policy areas, sectors and professional groups. These gaps hamper the opportunities of advising policy-makers on how to develop and govern a health workforce that is both quantitatively and qualitatively able to support the needs and demands being made on health systems as well as to implement policy reforms that allow health system performance and sustainability to improve. If we agree that there is ‘no health without a workforce’ [13], then the next step must be to close these knowledge gaps and advance problem-oriented research [21, 88]. The occupational structure of healthcare is inherently conservative, but various opportunities for change are currently emerging, among others, in the education systems, more integrated service and people-centred (persons, patients, users, populations) delivery models, new technologies, and new emergent roles of professional groups [9–11, 26, 44, 83, 89, 90]. These opportunities must be explored and aligned more systematically to enhance transformative powers.

Ultimately, producing knowledge is an important step, but on its own it is not enough. In order to build an integrated future health workforce, knowledge transfer, knowledge brokering and leadership must be developed in such a way that they solve problems and serve the needs of diverse health systems and populations. These complex demands cannot be addressed only within the current landscape of pilot studies and opportunity-driven ad-hoc responses to research calls. What is needed is a comprehensive research programme that connects the different disciplines and theoretical and methodological approaches involved in health workforce research. There is therefore an urgent need for building a health workforce as an independent, interdisciplinary and multi-professional field with better funding resources, new academic education programmes, comparative methodology, and knowledge transfer and leadership. This will help countries to build a people-centred health workforce.

## Abbreviations

EU: European Union
NCDs: Non-communicable Diseases
OECD: Organisation of Economic Co-operation and Development
